# The Degree of Branching of Serum IgG N-glycans as a Marker of Advanced Endometriosis

**DOI:** 10.3390/molecules29215136

**Published:** 2024-10-30

**Authors:** Ewa Maria Kratz, Katarzyna Sołkiewicz, Marcin Jędryka

**Affiliations:** 1 Department of Laboratory Diagnostics, Division of Laboratory Diagnostics, Faculty of Pharmacy, Wroclaw Medical University, Borowska Street 211A, 50-556 Wroclaw, Poland; katarzyna.solkiewicz@gmail.com; 2 Department of Oncology, Gynecological Oncology Clinic, Faculty of Medicine, Wroclaw Medical University, Hirszfeld Square 12, 53-413 Wroclaw, Poland; marcin.jedryka@umw.edu.pl

**Keywords:** immunoglobulin G, IgG glycosylation, advanced endometriosis, diagnostic markers

## Abstract

Endometriosis is a gynecological disease for which the diagnostics are difficult and often invasive; therefore, non-invasive diagnostic methods using sensitive and specific parameters present in easily available body fluid such as blood serum are needed for the detection of this disease. Our study aimed to answer the question of whether there are any differences between women with advanced endometriosis (AE), patients with gynecological diseases other than endometriosis (NE), and healthy women (control) in terms of the number of antennas of N-glycans from serum IgG. The degree of branching of IgG N-glycans was determined by a modified lectin ELISA with biotinylated lectin Con A (*Canavalia ensiformis* agglutinin) recognizing α-linked mannose, specifically reacting with biantennary N-glycans. The PHA-L/Con A ratio was calculated from the obtained N-glycan reactivities with Con A and PHA-L (*Phaseolus vulgaris* leucoagglutinin, specific to tri- and/or tetra-antennary N-linked glycans). The expression of Con A-reactive biantennary N-glycans in serum IgG was significantly lower in the control group than in the NE group (*p* = 0.045). The values of the PHA-L/Con A ratio were significantly higher in the NE group than in the AE and control groups (*p* = 0.019 and *p* = 0.022, respectively). The PHA-L/Con A ratio could be taken into account as a parameter helpful in the non-invasive diagnosis of advanced endometriosis, thus differentiating this disease from other gynecological diseases with an inflammatory background.

## 1. Introduction

Endometriosis is a disease characterized by the growth of endometrial tissue outside the uterine cavity, usually accompanied by pelvic pain and painful menstruation. It is a chronic inflammatory gynecological disease, often with non-specific symptoms; thus, the clinical diagnosis of the disease is very difficult [1]. The etiology of endometriosis is not fully understood and is multifactorial, with associated factors including genetic predisposition, the immune system, and sex hormones [2,3,4]. Until recently, laparoscopy with histological verification was used as the gold standard in endometriosis diagnostics; however, this method is invasive and involves the risk of complications for the patient. The ESHRE guideline from 2022 no longer supports laparoscopy as the gold standard for endometriosis diagnostics, and laparoscopy is currently only recommended for patients with negative imaging results and/or where empirical treatment was inappropriate or unsuccessful. However, members of the Endometriosis Guideline Core Group asserted that there is still an urgent need for more research into the most appropriate diagnostics, including laboratory diagnostics [5], especially given that one of the currently recommended gold standard approaches, 3D transvaginal ultrasound, does not guarantee the detection of the disease, even in its advanced stage of development, when the endometriotic lesions occur outside the measurement range of the device. Even though social awareness and interest in the problem of endometriosis in the scientific world has increased significantly in recent years, the non-invasive diagnosis of this disease, due to the lack of sensitive and highly specific markers, is still a great challenge. It has been reported that endometriosis is a disease accompanied by changes in the profile and degree of immunoglobulin G (IgG) glycosylation [6,7,8,9,10]. Immunoglobulin G is a glycoprotein most abundant in the blood (representing about 75% of serum immunoglobulins), and is involved in the pathogenesis and progression of many diseases. IgG glycans are essential for the proper activity of the immune system. Typical in the IgG glycosylation pattern is the presence of one biantennary N-linked glycan attached to asparagine 297 (Asn-297) of the crystallizable fragment (Fc region) [11,12], which consists of a constant heptameric core structure containing three mannose residues, four N-acetylglucosamine (GlcNAc) residues, and, additionally, may contain core fucose as well as bisecting GlcNAc. Moreover, the branching arms may have variable glycosylation patterns depending on the presence of terminal galactose, sialic acids, and antennary fucose [11,13,14,15]. The presence or absence of one sugar moiety in the N-glycan structure may stimulate or suppress the immune response [16]. In addition to Asn-297 of the Fc fragment, 15–20% of natural human IgG molecules contain N-linked oligosaccharides in the antigen-binding fragment (Fab region) [13,17,18]. The presence of sugar residues in a Fab usually increases the affinity of the antibody for the antigen. However, this depends on the hypervariable site to which the glycans are attached [19]. In our previous study, we showed significant differences in the reactivity of IgG isolated from serum (but not native serum IgG) with the *Phaseolus vulgaris* leucoagglutinin (PHA-L) [8], which binds to β1,6 branches of tri- and tetra-antennary oligosaccharides [20], between women with advanced endometriosis and patients with mild gynecological diseases in comparison to healthy women from the control group [8].

The present study aimed to check whether there are differences between patients with advanced endometriosis, women without endometriosis but with mild gynecological diseases, and a group of healthy women in terms of the relative reactivity of serum IgG N-glycans with Concanavalin A (Con A) specific to biantennary N-glycans. In addition, the ratio of lectin reactivity with three- and tetra-antennary glycans to reactivity with biantennary N-glycans in IgG (PHA-L/Con A) was analyzed in the context of its utility for the differentiation of the studied groups of women.

## 2. Results

**Figure 1:**
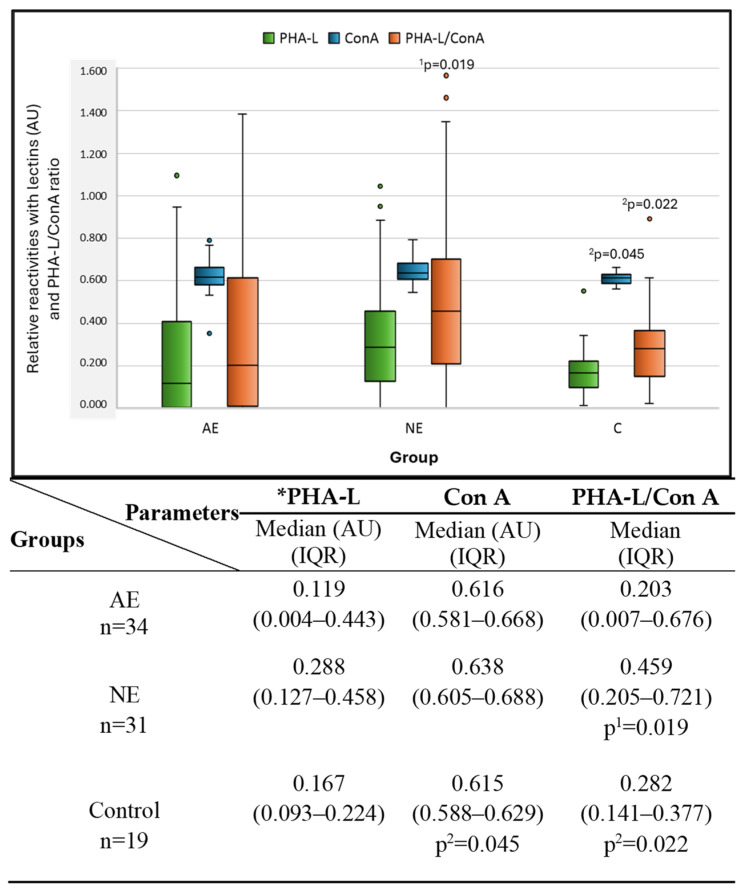
The values of relative reactivities of serum IgG N-glycans with * PHA-L, Con A, and PHA-L/Con A ratio. The results are expressed in absorbance units (AUs) as a median with an interquartile range (Q1–Q3). Serum IgG glycan reactivities with lectin were examined by lectin-ELISA and expressed in AU. Significant differences versus groups: ^1^ with advanced endometriosis (AE), ^2^ non-endometriosis (NE), and control—a group of healthy women. Con A—relative reactivity of IgG glycans with *Canavalia ensiformis* agglutinin; PHA-L/Con A—a ratio between IgG N-glycan reactivity with PHA-L and IgG N-glycan reactivity with Con A; PHA-L—*Phaseolus vulgaris* leucoagglutinin, n—number of patients. For lectin specificity, see the Materials and Methods section. * IgG N-glycan reactivity with PHA-L was previously determined by us and presented as mean ± SD (standard deviation) in an article by Sołkiewicz et al. [8]. Here, the obtained values are used for comparison with IgG N-glycan reactivities with Con A and the calculation of the PHA-L/Con A ratio. In the chart, the median is indicated as a line, colored boxes are 25–75% of results obtained, whiskers indicate min–max, and dots are outliers. A two-tailed *p*-value (probability value) of less than 0.05 was considered significant.

The relative reactivities of serum IgG glycans with biotinylated lectin, specific to biantennary N-linked glycans (Con A), and the ratio of PHA-L/Con A are presented in Figure 1.

**Figure 2:**
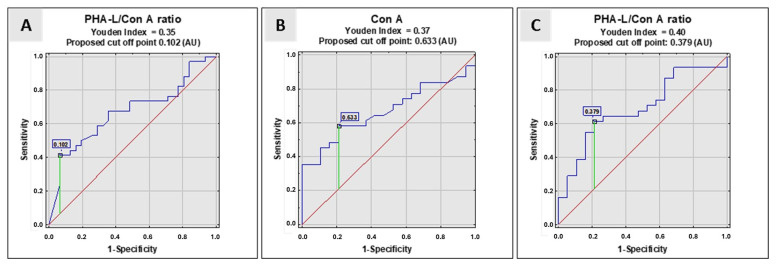
ROC curve analysis: (**A**)—PHA-L/Con A ratio in women with advanced endometriosis vs. non-endometriosis group, (**B**)—relative reactivity of IgG glycans with Con A in NE group versus healthy women, and (**C**)—PHA-L/Con A ratio for non-endometriosis women versus control group. Con A—relative reactivity of IgG N-glycans with *Canavalia ensiformis* agglutinin; PHA-L/Con A—a ratio between the reactivity of IgG N-glycans with PHA-L and their reactivity with Con A; PHA-L—*Phaseolus vulgaris* leucoagglutinin. For the specificity of Con A and PHA-L, see the Materials and Methods section. The reference line is marked in red, the receiver operating characteristics for the parameter in blue, and the cut-off point in green.

The expression of Con A reactivity with serum IgG glycans was significantly lower in the control group than in the NE group, but no differences were observed when the obtained results were compared to those received for women with advanced endometriosis. The PHA-L/Con A ratio was calculated based on the IgG N-glycans reactivity with biotinylated lectins PHA-L [8] and Con A. The PHA-L/Con A ratio was significantly higher in the NE group than in the AE and control groups. The verification of the clinical value of the examined parameters was based on the value of the area under the ROC curve (AUC). It can be defined as zero (AUC: 0–0.5), limited (AUC: 0.5–0.7), moderate (AUC: 0.7–0.9), and high (AUC: >0.9) [21]. Table 1 and Figure 2 present the results of the ROC curve analysis only for parameters for which the AUC was higher than or equal to 0.668.

## 3. Discussion

The composition of IgG N-glycans is well understood and is characterized by heterogeneity, showing a significant degree of variability within populations. The N-glycosylation pattern of IgG is influenced by both genetic and environmental factors, making it an important biomarker of overall human health. Changes in IgG glycosylation profile and degree have been observed in the course of various diseases [22]; they modulate IgG effector functions, thereby contributing to both the development and progression of the disease, representing both the predispositions and the functional mechanisms related to the pathology of the disease [23]. Our previous study showed that the development of advanced endometriosis and other gynecological diseases with an inflammatory background is accompanied by changes in the profile and degree of IgG glycosylation [6,7,8,9]. This study aimed to check whether, in patients with advanced endometriosis and women with other gynecological diseases with accompanied inflammation, compared with healthy women, in addition to the previously observed changes in IgG glycosylation, there are also changes in the values of the PHA-L/Con A ratio (IgG N-glycan reactivity with PHA-L to their reactivity with Con A) that would express the relationship between highly branched and biantennary N-glycans of IgG. Previously, we reported the presence of significant differences in the reactivity of serum IgG with PHA-L, but only for IgG isolates and not for native serum IgG, between women with advanced endometriosis, and patients with mild gynecological diseases in comparison to healthy women from a control group [8].

The relative reactivity of IgG N-glycans with Con A as well as the value of the PHA-L/Con A ratio significantly differentiated the group of women with gynecological diseases other than endometriosis from healthy women (control group). What is important for proper endometriosis diagnostics is that the PHA-L/Con A ratio also enables the differentiation of women with advanced endometriosis from the NE group of patients. To check the usefulness of the PHA-L/Con A ratio for the clinical differentiation of all three examined groups of women, a ROC curve analysis was carried out. It was concluded that the clinical value of the studied parameter was limited, but significant (AUC = 0.668, *p* = 0.0132 for AE vs. NE; AUC = 0.694, *p* = 0.01 for NE vs. C). The high specificity (93.5%) of the PHA-L/Con A ratio in the differentiation of the AE and NE groups is worth noting. This is precious information due to the serious difficulties in differential diagnosis between AE and NE groups, as endometriosis is a disease without specific symptoms and is frequently confused with other gynecological diseases. However, we are aware of certain limitations of our research, which may indicate that the results obtained and the conclusions drawn from them are not fully satisfactory. When planning further studies, it will be necessary to increase the number of participants in the studied groups to check whether the low sensitivity of the PHA-L/Con A ratio in differentiating the AE and NE groups and the lack of significant differences in the values of this parameter between the AE group and the group of healthy women are caused by too few study participants.

The PHA-L/Con A ratio allows us to observe that, in the case of the NE group, there is a noticeable change in the proportion between the expression of highly branched IgG N-glycans and biantennary N-glycans (about 1:2) compared to the group of women with advanced endometriosis (about 1:5) and healthy women (about 1:3). Taking into account that serum IgG is characterized by the presence of mainly biantennary glycans, the value of PHA-L/Con A ratio observed in these studies for the non-endometriosis patients may be a characteristic feature of this group, additionally indicating the potential diagnostic usefulness of this parameter for AE and NE differentiation. Interestingly, as documented in our previous study, the observed trend does not seem to be dependent on IgG concentrations in the NE and AE groups, as the differences in serum IgG concentrations between these groups were not significant [24]. An increase in N-glycan branching observed for acute-phase proteins is a known marker of chronic inflammation, and it the fact that some analogies may exist in the case of the expression of highly branched N-glycans of IgG in mild gynecological diseases (NE group) that are accompanied by chronic inflammation cannot be excluded. It is also interesting that both PHA-L and Con A reactivity with IgG N-glycans does not allow the differentiation of the AE and NE groups—only the value of the PHA-L/Con A ratio allows for such a possibility, which may indicate that the expression of a single type of IgG glycans is insufficient for differentiating the examined diseases, but their mutual expression fulfills this task. Based on our previous analyses of serum IgG relative reactivities with sialo-specific lectins in advanced endometriosis and other mild gynecological diseases [6], we observed the presence of weak negative correlations between the reactivities of isolated serum IgG with PHA-L and its reactivities with MAA and SNA [8]; however, such correlations were not observed for native serum IgG. Summarizing the results of native serum IgG N-glycan analysis, it can be concluded that, in AE women, when compared to NE patients, the significantly decreased PHA-L/Con A ratio is accompanied by the significantly increased expression of terminal galactose [6] and core fucosylation [7]. The α2,6 sialylation of IgG was also significantly higher in AE than in NE women but the lectin-ELISA did not enable us to determine whether the detected sialic acid is a part of N- or O-glycans of native serum immunoglobulin G.

It should be taken into account that the IgG glycosylation analysis provided in this and previous studies included glycans present both in the Fc and Fab regions of IgG, without any differentiation between them; thus, it is not possible to determine in which of the IgG regions the observed glycosylation changes occur. Crystallographic studies conducted by Deisenhofer in 1981 [25] showed that the oligosaccharides attached to the Asn 297 of IgG Fc fragments are mainly located internally in the secondary/tertiary structure of the molecule. This was confirmed by Huang et al. [26], who reported that the internal location of the β2 residue of Man α1 in the native Fc region was inaccessible to Con A regardless of glycoforms. Based on the above information, it can be concluded that the changes in reactivity of IgG glycans with Con A most probably concern glycans attached to variable antibody fragments; however, this hypothesis requires further research on glycosylation of specific regions of the IgG molecule. The analysis of glycoprotein glycosylation using the lectin-ELISA test can serve as a model reflecting the accessibility of glycoprotein glycans for their endogenous ligands, and our studies on the degree of branching of serous IgG N-glycans in the course of advanced endometriosis also show that considering the participation of IgG glycans in many pathophysiological processes and the accessibility of IgG glycans for specific ligands is crucial.

From a clinical point of view, this study found that serum IgG N-glycan branching analysis can potentially be used as an efficient, non-invasive tool for advanced endometriosis detection. The clinical value of this novel biomarker might be of great significance if confirmed in further prospective validated studies that compare this technique with the present clinical approaches of sophisticated, high-resolution imaging (3D transvaginal ultrasound and/or magnetic resonance) followed by minimally invasive surgical verification.

## 4. Materials and Methods

### 4.1. Patient Samples

Serum samples from premenopausal women with diagnosed advanced endometriosis (AE; n = 34, mean age: 35 ± 7 years), as well as from a group of women without endometriosis (NE; non-endometriosis; n = 31, mean age: 38 ± 8 years) were collected at the Department of Oncological Gynecology, Wroclaw Comprehensive Cancer Center, Lower Silesian Oncology, Pulmonology and Hematology Center, Poland. The patients underwent surgical interventions, mainly laparoscopic, and were classified into the proper group after histological verification. Women with advanced endometriosis were classified on the extent and severity of the disease according to the revised American Fertility Society (rAFS) classification. The non-endometriosis group was histologically confirmed with a benign ovarian cyst, with severe dysplasia—CIN 3 (cervical intraepithelial neoplasia grade 3) or leiomyomas. The control group consisted of healthy women, with no symptoms or history associated with endometriosis, non-pregnant, and without any gynecological diseases. All participants of the study had a comparable body mass index (median BMI for all three groups was in the range of 24–26). Serum samples from healthy women (C; control group; n = 19, mean age: 40 ± 8 years) were collected at the Department of Laboratory Diagnostics, Wroclaw Medical University (with approval from Bioethics Committee No. KB-117/2020). The control group consisted of non-pregnant, premenopausal women without any gynecological problems and symptoms of illness, with no disease history connected with endometriosis. Before starting the study, all participants gave written and informed consent. The study was conducted in agreement with the Helsinki-II declaration, and the protocol was approved by the Bioethics Human Research Committee of Wroclaw Medical University (Permission No. KB-293/2016, KB-719/2018 and KB-180/2021).

### 4.2. Lectin ELISA

The degree of branching of the IgG N-glycans was determined by a modified lectin-based ELISA with biotinylated lectin Con A (Vector Laboratories Inc., Burlingame, CA, USA) that recognizes α-linked mannose, specific for biantennary-type N-linked glycans. The PHA-L/Con A ratio was calculated based on the obtained values of relative reactivities of IgG N-glycans with PHA-L (selective for tri- and/or tetra-antennary N-linked glycans) (Vector Laboratories Inc., Burlingame, CA, USA) [8] and Con A. The IgG concentrations in the analyzed sera, necessary for the calculation of the IgG amount for the lectin ELISA, were estimated using the turbidimetric method described previously by Kokot et al. [24]. The lectin ELISA test was performed according to the procedure described by us previously [6] with slight modifications. In short, microtiter plates (Nunc MaxiSorp, Thermo Fisher Scientific, Glostrup, Denmark) were incubated with 0.01 mg/mL protein G solution (Abcam, Waltham, MA, USA) in 10 mM TBS at pH 7.4 (2 h, 37 °C, and kept at 4 °C overnight). Next, the plates were coated with serum diluted with 10 mM TBS-T 0.1% (pH 7.4) in the amount of 500 ng IgG in 50 µL solution per well, and incubated (3 h, 37 °C). In the next step, the plates were incubated for 90 min at 37 °C with biotinylated lectin Con A diluted at a ratio of 1:250 with 10 mM TBS-T 0.1%. Next, the plates were incubated with phosphatase-labeled ExtrAvidin for 30 min at 37 °C, and then the phosphatase reaction was developed at 37 °C with a substrate, p-nitrophenyl-phosphate. The reaction was stopped with 100 µL of 1 mM NaOH per well, and the absorbance was measured at 405 nm with a reference filter λ = 630 nm, using a Mindray-96A microplate reader (Shenzhen Mindray Bio-Medical Electronics Co., Shenzhen, China). After each incubation step, the plates were extensively washed with 300 µL 10 mM TBS-T 0.1%, pH 7.4. All samples were analyzed in duplicate. The background absorbances (blank samples) were measured for samples containing all reagents, except the biological material, which was replaced with 10 mM TBS-T 0.1%, pH 7.4. The relative reactivities of IgG glycans with lectin were expressed in absorbance units (AUs).

### 4.3. Statistical Analysis

Statistical analysis was performed using the statistical software STATISTICA 13.3PL (StatSoft Inc., Tulsa, OK, USA). The values obtained for the relative reactivities of IgG N-glycans with lectins Con A and PHA-L were presented as median with interquartile range (Q1–Q3). According to a Shapiro–Wilk W test, the values did not fit a normal distribution; thus, the nonparametric Mann–Whitney U test was used to determine the differences among the groups. The diagnostic significance of the determined parameters was analyzed using receiver operating characteristic (ROC) curves.

## 5. Conclusions

Studies proposing a non-invasive, sensitive, and specific diagnostic marker that allows clinicians to perform the rapid diagnosis of endometriosis, as well as enabling the differentiation of this disease from other benign gynecological diseases, are in the spectrum of interests of many scientists. The parameters proposed in the present study are the degree of expression of biantennary N-glycans on serum IgG, which helps to distinguish women suffering from benign gynecological diseases other than endometriosis from healthy women; and, what is especially important, the PHA-L/Con A ratio, which differentiates patients with advanced endometriosis from women with gynecological diseases other than endometriosis. However, taking into account the limitations of our study resulting from the low number of study participants, future research should determine whether increasing the number of subjects participating in the study will improve the sensitivity of the PHA-L/Con A ratio in differentiating between AE and NE groups, and whether the values of this parameter differ significantly between the AE group and the control group of healthy women. Nevertheless, the results obtained in the present study provide a good starting point for further research on the diagnosis of endometriosis at an early stage of development, as well as for understanding the mechanisms responsible for the progression of this disease. It should be underlined that the potential for the use of IgG N-glycosylation alterations as novel biomarkers for the identification of gynecological disease predisposition and progression, as well as for diagnosis, prognosis, and response to therapy, cannot be ignored.

## Figures and Tables

**Table 1:** ROC curve analysis of IgG relative reactivity with Con A, and PHA-L/Con A ratio.

Lectin	AUC	AUC with 95% Confidence Interval	Cut-Off Point	Sensitivity	Specificity	*p*-Value
AE vs. NE						
PHA-L/Con A	0.668	0.535–0.802	0.102	0.412	0.935	0.0132
NE vs. C						
Con A	0.670	0.522–0.817	0.633	0.581	0.789	0.024
PHA-L/Con A	0.694	0.546–0.842	0.379	0.613	0.789	0.01

The table only presents the results of the ROC curve analysis for which the AUC value was higher than 0.668. Con A—relative reactivity of IgG N-glycans with *Canavalia ensiformis* agglutinin; PHA-L/Con A—a ratio between IgG N-glycan reactivity with PHA-L and IgG N-glycan reactivity with Con A; PHA-L—*Phaseolus vulgaris* leucoagglutinin. For lectin specificity, see the Materials and Methods section. Significant differences were accepted for a two-tailed *p*-value (probability value) of less than 0.05.

## Data Availability

Data are available from the corresponding author upon reasonable request.
